# Thinking outside the box: students positive about visionary elective curricula in medical school

**DOI:** 10.3205/zma001515

**Published:** 2021-11-15

**Authors:** Sven Olaf Rohr, Ameli Gerhard, Felicitas Schmidt, Julia Eder, Lukas Salvermoser, Konstantinos Dimitriadis, Martin R. Fischer

**Affiliations:** 1 LMU Munich, University Hospital of Munich, Institute for Didactics and Training Research in Medicine, Munich, Germany; 2 LMU Munich, University Hospital of Munich, Neurological Clinic, Munich, Germany

**Keywords:** medical education, interdisciplinary studies, self-directed learning, creativity, visionary medicine, medical humanities

## Abstract

**Objective:** Space for personality development as well as for the development of critical, creative and interdisciplinary thinking is rarely found in medical curricula in Germany. To be prepared for the challenges of modern medicine, future physicians need a visionary mindset. The aim of this study is to determine the need for teaching such content among medical students in the context of visionary elective curricula and to examine these with regard to the desired topics and organizational structure.

**Methods:** This is a cross-sectional study with 236 medical students from all semesters of the Ludwig-Maximilians-University Munich. The survey consists of 50 questions and includes single choice, multiple choice, matrix questions, open-ended questions and Likert scales. Responses were examined using descriptive statistics and compared parametrically in sub-aspects.

**Results:** Three-quarters of respondents would like to see curricular content on interdisciplinary interfaces with other disciplines. A suitable framework for this is seen by 87% of the respondents in a visionary elective curriculum. Students would like to see a broad range of specific content such as global health, politics, business, and computer science. The majority of respondents would like to see 1 unit of instruction per week and would participate in an appropriate program. Such an offering would promote creative (53.6%), critical (63.7%), and interdisciplinary thinking (69.0%) and train to become better physicians (87%).

**Conclusion:** Participants in this study are positive toward the introduction of visionary content in medical school. Faculties should build visionary elective curricula according to the graduate profile requirements of the new NKLM 2.0 to make medical education sustainable.

## Zusammenfassung

**Zielsetzung: **Platz für Persönlichkeitsbildung sowie für die Entwicklung kritischen, kreativen und interdisziplinären Denkens findet sich nur selten in medizinischen Curricula in Deutschland. Um auf die Herausforderungen der modernen Medizin vorbereitet zu sein, brauchen angehende Ärztinnen/Ärzte eine visionäre Geisteshaltung. Ziel der Studie ist es, den Bedarf für die Vermittlung solcher Inhalte unter Medizinstudierenden im Rahmen von visionären Wahlcurricula zu ermitteln und diese hinsichtlich der gewünschten inhaltlichen und organisatorischen Struktur zu untersuchen.

**Methodik: **Es handelt sich um eine Querschnittstudie mit 236 Medizinstudierenden aus allen Semestern der Ludwig-Maximilians-Universität München. Die Umfrage besteht aus 50 Fragen und beinhaltet Single-Choice, Multiple Choice, Matrixfragen, offene Fragen und Likert-Skalen. Die Antworten wurden mittels deskriptiver Statistik untersucht und in Teilaspekten parametrisch verglichen.

**Ergebnisse: **Dreiviertel der Befragten wünschen sich curriculare Inhalte zu interdisziplinären Schnittstellen mit anderen Fachrichtungen. Ein geeigneter Rahmen dafür wird von 87% der Befragten in einem visionären Wahlcurriculum gesehen. Die Studierenden wünschen sich ein breites Angebot spezifischer Inhalte wie Global Health, Politik, Wirtschaft und Informatik. Die Mehrheit der Befragten wünscht sich einen Zeitumfang von 1 Unterrichtseinheit pro Woche und würde an einem entsprechenden Programm teilnehmen. Ein solches Angebot würde das kreative (53,6%), kritische (63,7%) und interdisziplinäre Denken (69,0%) fördern und zu besseren Ärztinnen/Ärzten ausbilden (87%).

**Schlussfolgerung: **Die Teilnehmenden dieser Studie sind gegenüber der Einführung visionärer Inhalte im Medizinstudium positiv eingestellt. Fakultäten sollten entsprechend der Anforderungen des Absolventenprofils des neuen NKLM 2.0 visionäre Wahlcurricula aufbauen, um die Ausbildung von Ärztinnen und Ärzten zukunftsfähig zu machen.

## 1. Introduction

### 1.1. Challenges for future physicians

The mission of medical school is to prepare physicians for future practice. Physicians are tasked with maintaining and, if possible, restoring the health of the population [https://www.gesetze-im-internet.de/_appro_2002/BJNR240500002.html]. Little room is given to personality development [1], [2]. In a profession that deals primarily with people, reflection and a basic understanding of society and the culture in which one practices is essential [3]. Currently, medicine and society are undergoing accelerating change. Technological innovation, digitalization, climate change, and the COVID-19 pandemic are just a few challenges facing medicine and society. A physician is at the center of society with medicine and must continuously adapt to the ongoing changes in the world in order to guarantee the best possible care for the population. Mere professional knowledge therefore does not seem to be sufficient. In times of Hippocrates, physicians therefore developed additional competencies, such as in philosophy. A physician in the 21^st^ century also needs the ability to deal critically, creatively and interdisciplinarily with his capabilities and his role in society. These skills should therefore also be reflected in medical studies. In the Anglo-American world, the teaching of these skills has long been given space with the so-called Medical Humanities, where they are an integral part of many curricula [https://med.stanford.edu/medicineandthemuse/ProgramLinks/OtherPrograms.html]. A few years ago, the first German professorship for this topic was established at the Charité Berlin [4]. The Julius-Maximilians-University of Würzburg offers with the voluntary *“Philosophicum”* the possibility to question fundamental views on medicine [5], [6]. With the program *“Lettered Medical Education”*, the Technical University of Munich has created a voluntary program that aims to prepare future physicians for their human responsibility and to promote the examination of their own personality [7], [8]. The positive influence of Medical Humanities on the personality formation and resilience of medical students and physicians as well as on the performance in multiple choice exams could be shown in several studies [9], [10], [11]. Similarly, the study of philosophy and other interdisciplinary content has long been called for by various experts [3], [6]. Since the 2000s, the digitalization of central areas of the working world has also found its way into healthcare and medicine and promises many opportunities [12], [13]. It is essential for future physicians to develop an understanding of the perspective of information technology [14]. In the area of digitization, there has been at best sporadic curricular content nationwide [15], [16], [17]. An overview of the individual offerings at German faculties can be found in the research by Aulenkamp et al. (2021) [18].

At most German medical faculties, there is a large discrepancy between the requirements for future physicians and the actual training concepts [8]. In the 11^th^ Student Survey of the Working Group on Higher Education Research at the University of Konstanz it is stated: “According to the majority of the students surveyed, far too little value is placed on: developing their own focal points of interest; analyzing complex issues; criticizing teaching opinions; participating in discussions in courses; applying what they have learned to practical issues; taking an interest in social and political issues from the perspective of the subject; dealing with ethical issues; applying research methods independently” [1]. This situation has also been confirmed in subsequent student surveys [2]. However, there has not yet been a systematic investigation of how medical students perceive this issue.

#### 1.2. The physician as visionary

In order to define the physician role model and make it usable for medical education, various approaches have been pursued in the past and attempts have been made to make them usable for medical education. The CanMEDS roles represent a model that has been widely used for this purpose [19]. They were adapted to German-speaking countries for the creation of the NKLM 2015 *(Nationaler Kompetenzbasierter Lernzielkatalog Medizin)* and linked to competence-oriented teaching [http://www.nklm.de]. In recent years, it has been noticed – especially in the course of digitization – that it is difficult to map innovative, system-transcending ideas or even learning objectives within the existing role definitions. 

**Figure 1:**
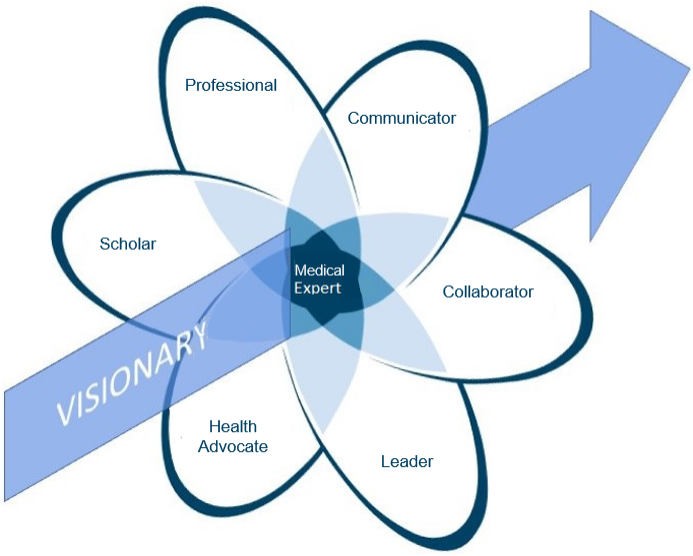
llustration of the six CanMEDs roles with the addition of the visionary system transcendent axis (adapted from [19], modified from [26]).

Federal policy has responded to the aforementioned demands on future physicians. First reforms such as the *Masterplan Medizinstudium 2020 (MM 2020)* have been underway since 2015 [20]. The German Council of Science and Humanities *(Wissenschaftsrat)* demands that in addition to 75% (4500 h) core curriculum, 25% (1500 h) elective curriculum should be offered to allow individual focus training in addition to teaching basic medicine competencies [21]. In the course of the further development of the *NKLM 2.0* [22], [http://www.nklm.de] demanded by MM 2020 and promoted by *IMPP (Institut für medizinische und pharmazeutische Prüfungsfragen), MFT (Medizinischer Fakultätenta), bvmd (Bundesvertretung der Medizinstudierenden in Deutschland e.V.)* and other associations, competencies of the „visionary axis" were therefore demanded for the first time in the graduate profile (Chap. IV.2.8). These are integrated into the corresponding EPAs (entrustable professional activities) or nested EPAs, which form the basis for the learning objectives. The “visionary axis” is a newly introduced concept in the German training landscape, accordingly many aspects of the concept are still insufficiently investigated. In an international comparison, some contents overlap with the field of Medical Humanities discussed above and have been studied in its context [9], [23], [24]. However, the concept of the visionary axis goes further and includes other system-transcending dimensions. In the graduate profile of the NKLM, the visionary axis is defined as follows: “The genuine task of lifelong learning of physicians is the formation of a critical way of looking at things and the constructive and future-oriented working towards innovations and meaningful changes. Learning is understood as a productive-creative, activating, context- and socially-sensitive process. In the personal confrontation with the reality of patient care, students acquire skills for critical evaluation as well as the competencies and willingness to help shape this reality in the sense of a sustainable, patient- and person-centered medicine. In doing so, they know how to deal creatively with uncertainties, understand diversity and individuality as enrichments, and connect their daily actions in the knowledge of the past and with orientation towards the future” [25]. The integration of the visionary axis into the *NKLM 2.0* was partly done on the initiative [26] of the *bvmd*, which had called for an extension of the previous CanMEDS roles to include a visionary axis (see figure 1 (Fig. 1)).

**Figure 2:**
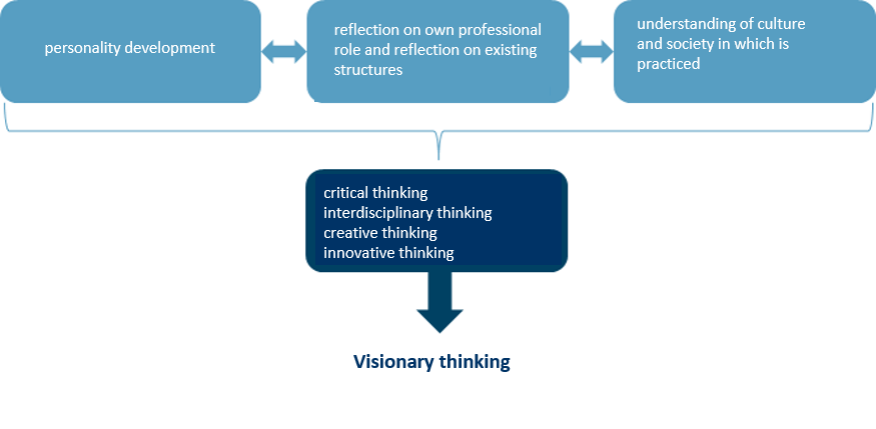
Goals and core elements of visionary thinking

Visionary (elective) curricula are one way of implementing this new curricular content in practice. They should expand the medical curriculum to include all those topics and competencies that are essential for personality development, reflection on one’s own professional role, and for a better understanding of societal structures (see figure 2 (Fig. 2)). This should promote critical, interdisciplinary and creative thinking. Currently, there is no medical faculty in Germany with an elective curriculum that is visionary in this sense. The described characteristics of the visionary axis include a variety of skills and abilities, some of which are difficult to operationalize. Therefore, it is necessary to investigate an evaluation as well as a prioritization of content from the perspective of medical students. In addition, their assessment of the relevance of individual skills for their studies and everyday professional life should be analyzed.

#### 1.3. Aims of the study

The aim of the present study is to investigate the student perspective regarding a visionary elective curriculum in terms of the *NKLM 2.0*. This orienting study’s goal is to map in a survey among medical students of the Ludwig-Maximilians-University of Munich (LMU) whether the introduction of a visionary elective curriculum is desired from the students’ perspective, which topics are assessed by the students as relevant in terms of content and to record which organizational framework conditions (e.g. temporal scope of the elective curriculum) would be ideal from the students' perspective.

## 2. Methods

### 2.1. Preparation of the survey and questionnaire design

In consultation with the ethics committee of the LMU Munich, there are no ethical-legal objections to the study. In creating the survey, the results of orienting focus group interviews were accessed. In these, on the one hand, a general interest of medical students in a visionary elective curriculum was established, on the other hand, the preferences and interests of the students regarding possible contents of the curriculum were surveyed. The final survey consisted of 50 questions. Preceding the survey were introductory texts explaining the visionary elective curriculum and how the survey was conducted. The questions were two matrix questions, ten single-choice questions, three multiple-choice questions, nine open-ended questions (two with free text), and 24 Likert scales. The decision was made to use 6-point Likert scales. This was done deliberately not to provide survey participants with a neutral choice, but at the same time to provide a sufficiently differentiated range of response options [27]. Depending on the question, five different poles were used (very positive/very negative; very supported/not supported; very well covered/not covered; very interested/not interested; strongly agree/strongly disagree). The survey was created using the software EvaSys (Lüneburg, Germany). A pilot was conducted by initially administering the questionnaire to 10 subjects. This resulted in an average completion time of 4:30 minutes and no technical difficulties in completion. The results of the subjects were not included in the survey results.

#### 2.2. Conduction of the study

250 questionnaires were distributed to medical students of the Ludwig-Maximilians-University (LMU) within two weeks. Potential participants were approached personally before the start or after regular, curricular events to avoid selection bias. Participants were from both preclinical (N=63) and clinical study sections (N=173), ranging from first to 12^th^ semester (see primary data for details, attachment 1 ). The response rate was 96.8% (voluntary participation). The response rate was based on the proportion of questionnaires received to questionnaires distributed. As an inclusion criterion, only LMU medical students were eligible to complete the survey. Six questionnaires had to be excluded ex-post because the respective participants indicated that they had not completed the questions truthfully or were not students of LMU.

#### 2.3. Statistical analysis

The statistical analysis was performed with the software IBM SPSS 25 (Armonk, USA). For the creation of relative frequency values, only the answered questions were evaluated as the population in each case. A descriptive analysis of demographic and thematic preferences was performed. For the evaluation of the students' assessment, Likert rating 1 and 2 were each evaluated as positive expression, Likert rating 5 and 6 as negative expression. Likert rating 3 was evaluated as “tending to negative”, Likert rating 4 was evaluated as “tending to positive”. The free texts were screened by two authors and sorted into groups according to content. Single-factor ANOVAs and t-tests were used for parametric comparisons. Distributional hypotheses were tested by nonparametric tests. A significance level of α=0.05 was assumed for all tests.

## 3. Results

### 3.1. Demographics of the participants

**Figure 3:**
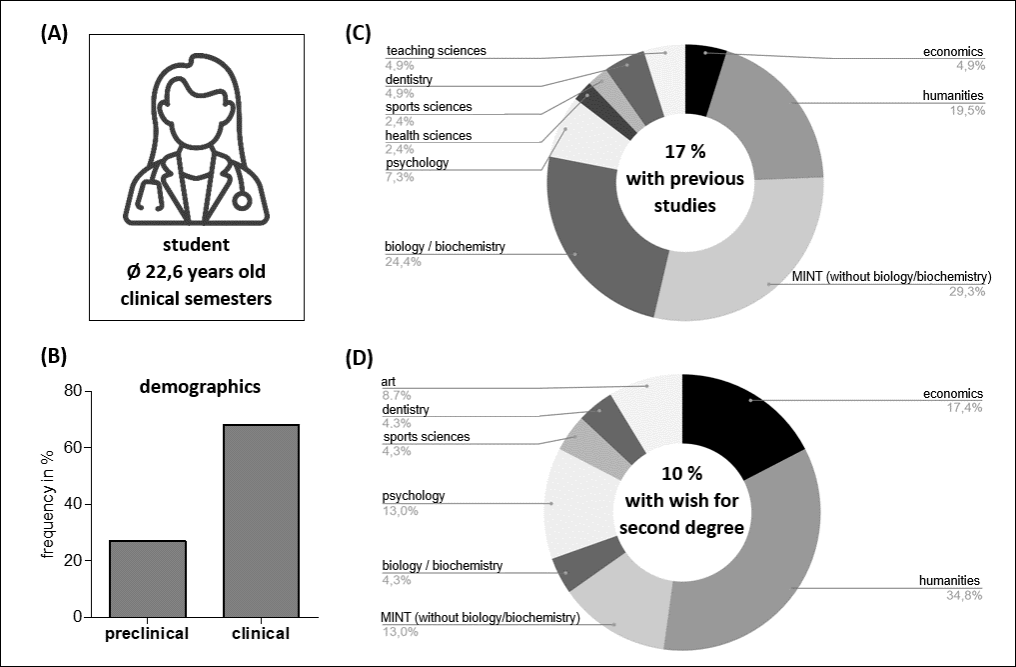
Demographics. (A) The average study participant is female, 22.6 years old, and in the clinical study semester. (B) Respondent's affiliation with study section. *Plot: relative frequencies, N=263; in exempt semester: 2, in practical year: 1, not specified: 9.* (C) Proportionate distribution of fields of study taken by respondents before medical school. *Plot: absolute and relative frequencies, N=41.* (D) Subject preferences of respondents for a desired parallel second degree. *Plot: absolute and relative frequencies, N=23.*

Questionnaires from N=236 students were included in the survey analysis (see figure 3 (Fig. 3)). The average participant was 22.6 years old, female (66.0%), and in the clinical study section (68.0%). Of all survey participants, 17.0% reported that they were enrolled in another field of study prior to entering human medicine. 2.5% of students were concurrently enrolled in another field of study at the time of the survey. 21.2% mentioned having already completed professional training. 10.0% of students expressed interest in pursuing a second degree. Figure 3 (Fig. 3), items C and D show the study preferences that were indicated for a possible second degree program.

#### 3.2. Evaluation of the current study situation compared to visionary elective curricula and topic focus

**Figure 4:**
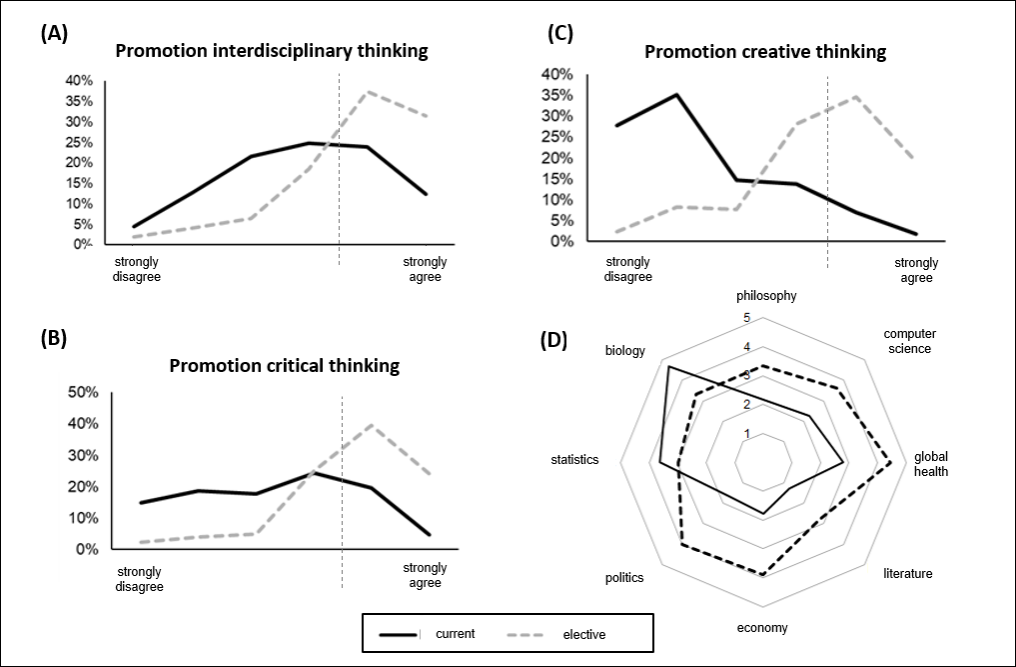
Comparison of current curriculum (as-is) with visionary elective curricula in terms of promoting (A) interdisciplinary, (B) critical, and (C) creative thinking. *Plot: relative frequencies from 6-point Likert scales, N=232 (interdisciplinary), N=230 (critical), N=231 (creative).* (D) Assessment of current coverage of extracurricular interfaces in the curriculum and desired coverage in a visionary elective curriculum. *Plot: mean values from 6-point Likert scales, N=236.*

The majority of students (62.8%) indicated that the current medical school curriculum did not promote much creative thinking (Likert 1-2). 53.6% of students expected that a visionary elective curriculum would promote creative thinking (Likert 5-6). One-third of students (33.5%) felt that critical thinking was not promoted much in the curriculum (Likert 1-2). 63.7% of students expected that a visionary elective curriculum would promote critical thinking (Likert 5-6). 18.5% of the participants saw interdisciplinary thinking as little promoted by the course of study (Likert 1-2). A significant promotion would also be expected by 69.0% of the students with regard to interdisciplinary thinking (see figure 4 (Fig. 4)). With regard to the subject orientation of the study program, most students saw topics such as biology (Likert mean: 4.67) and statistics (Likert mean: 3.61) as already being covered to a sufficient extent. Other topics such as global health (Likert mean: 2.80), computer science (Likert mean: 2.25), philosophy (Likert mean: 2.16), economics (Likert mean: 1.78), politics (Likert mean: 1.68), or literature (Likert mean: 1.30) appear to be covered rather less (see figure 4 (Fig. 4), Item D). In terms of topic orientation of visionary elective curricula, respondents were strongly inclined toward topic interfaces with global health (Likert mean: 4.45), politics (Likert mean 4.04). Respondents tended to be inclined toward topic interfaces with economics (Likert mean: 3.90) and computer science (Likert mean 3.63). Students tended to be averse to addressing topic interfaces with biology (Likert mean: 3.35) and philosophy (Likert mean: 3.32). Students were strongly averse to statistics (Likert mean 2.98) and literature (Likert mean 2.79). Other topics suggested in free text were ethics (N=4), psychology, especially with interviewing & communication (N=8), law (N=5), languages & rhetoric (N=6), history (N=5), practical skills (N=3), sports (N=3), nursing and physical therapy (N=2), engineering and technology (N=2), and with one mention each: music, dentistry, general knowledge, arts & culture, research, establishment, and alternative medicine. Pre-clinical and clinical students did not differ significantly (data not shown).

#### 3.3. Evaluation of visionary elective curricula. 

**Figure 5:**
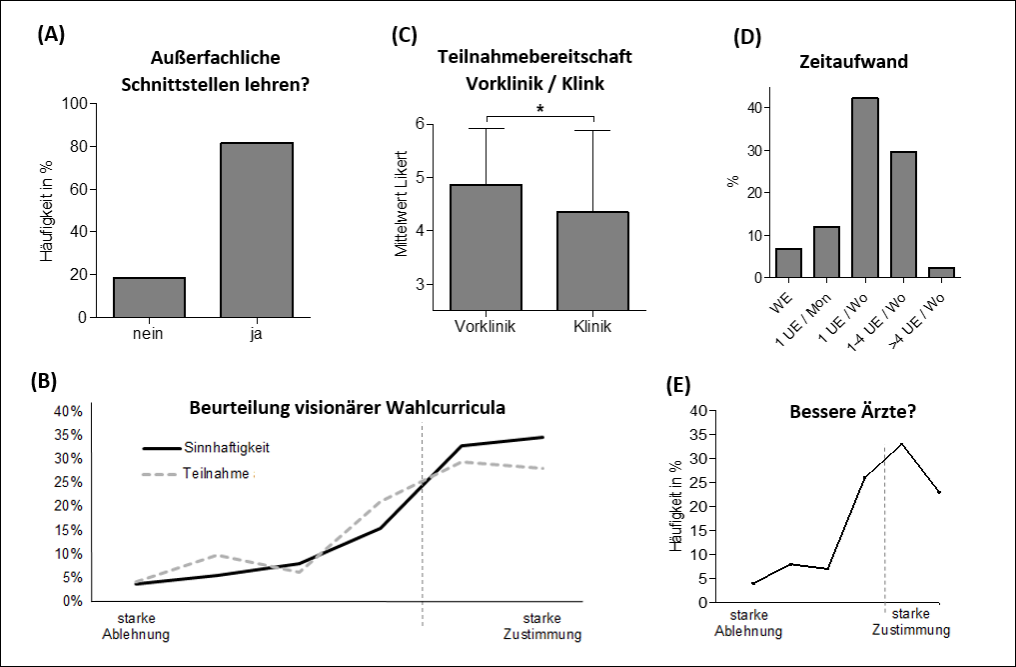
(A) Should interfaces of medicine with non-medical specialties be taught? *Plot: relative frequencies, N=215 *(B) Assessment of the usefulness of a visionary elective curriculum *(N=214)* and willingness to participate in such a program *(N=211).* *Plot: relative frequencies from 6-point Likert scales. *(C) Comparison of willingness to participate in such a program between preclinical and clinical settings. Plot: means from 6-item Likert scales, N=210, ANOVA, * = p<0.05. (D) Desired number of teaching units (UE) for visionary elective curricula in different time periods. *Plot: Relative frequencies from single-choice questions, N=206, WE=1 weekend course in semester, Mon=month, Wo=week.* (E) Do visionary elective curricula make students better physicians? *Plot: relative frequencies of a 6-point Likert scale, N=216.*

Overall, 81.6% of students supported the promotion of teaching thematic interfaces with non-medical specialties (see figure 5 (Fig. 5), item A). There was no significant difference between the wishes of preclinical (mean: 1.10; SD: 0.31; scale: “yes”=1.0; “no”=2.0) and clinical (mean: 1.22, SD: 0.41) students. A visionary elective curriculum was considered useful by 67.3% (see figure 5 (Fig. 5), item B, Likert 5-6). 9.3%, on the other hand, could not see any added value in such an additional offering (Likert 1-2). More than half (58.3%) of the students stated that they would like to participate in a visionary elective curriculum (Likert 5-6). 21.3% expressed at least a positive tendency (Likert 4) to want to take advantage of this offer. Students in the preclinical study section (average Likert rating: 4.9; SD: 1.1) would participate in such an elective curriculum significantly more often (p=0.02) than students in the clinical study section (average Likert rating: 4.4; SD: 1.5). Regarding the time commitment, most students (42.2%) were in favor of one teaching unit (UE) per week of 45 min (see figure 5 (Fig. 5), item D). 

More than half of the students (56%, Likert 5-6) felt that a visionary elective curriculum could make them better physicians. A quarter (25.8%) expressed at least a positive tendency (Likert score: 4). In free-text responses categorized by the authors, this was explained by the increase in general education (55.2%), the promotion of critical and creative thinking (25.4%), and expansion of non-medical expertise (3.0%) and personality development (1.5%).

## 4. Discussion

This paper is the first study to examine students’ perspectives on the new “visionary axis” in competency-based curricula [25] called for in the *NKLM 2.0*. Visionary curricula are designed to foster a visionary mindset in order to educate visionary physicians who can meet the societal challenges of today and tomorrow with critical, interdisciplinary, and creative thinking [26]. The results of this study should provide food for thought to build visionary curricula in Germany. It is a study with 236 participants from clinical and preclinical semesters. The high response rate of >96% could be achieved by conducting the survey in randomly selected, attendance-required face-to-face courses with high staffing. In this way, we felt that a possible selection bias for students who were already interested and would be more likely to participate in a relevant survey was counteracted. Orienting focus group interviews with students conducted in advance allowed us to generate hypotheses for the final survey. Despite a small pilot sample size of 10 students, basic information about the feasibility and comprehensibility of the questions was obtained. The fact that the study was conducted at only one university means that it can only be partially transferred to other universities in Germany. In particular, it must be taken into account that significantly more students in the clinical study section participated in the survey. Nevertheless, it should be pointed out that the image of the “better doctor” is difficult to operationalize. This image is influenced by multiple aspects in the physician's understanding of his/her role. The *NKLM 2.0* also addresses this issue in Chapters III and IV [http://www.nklm.de]. In this article, in addition to examining the physician ideal in the pilot, the definition of “good physician” was further queried in the primary survey to capture an understanding of individual perceptions of this ideal among students. Responses to this can be found in the primary data in attachment 1 .

Based on the results on content aspects, we propose that a visionary elective curriculum be established as a longitudinal course in medical school. The majority of respondents favored 1 UE per week of 45 min. According to the respondents, such a course would have very great potential to promote creative, critical, and interdisciplinary thinking skills. This is in line with the goal of visionary curricula and is compatible with the requirements for individual focus required by the German Council of Science and Humanities [19].

Respondents would like to see a range of specific content to choose from (see figure 4 (Fig. 4)). A modular structure of the elective curricula with a possible internal credit system would therefore be advantageous. Already in the 2015 version of the *NKLM*, there are some learning objectives that could be mapped to a visionary curriculum, but are currently taught only sparsely [http://www.nklm.de]. Such learning objectives have become even more numerous in the new *NKLM 2.0* [http://www.nklm.de]. Visionary elective curricula would provide the framework to cover these new learning objectives in the curriculum. In the long run, prospective accompanying research is useful to verify whether the teaching of a visionary mindset is indeed achievable through appropriate curricula.

## 5. Conclusions

The present work is a cross-sectional study to survey the need for a voluntary visionary elective curriculum among students at LMU Munich. 75.0% of the students surveyed would like to see curricular content on interdisciplinary interfaces with other (non-medical) disciplines. This would promote creative, critical, and interdisciplinary thinking. 67.3% of respondents see visionary elective curricula as a suitable framework for this. The majority of respondents would like to see a timeframe of 1 UE/week. In terms of content, global health, politics, economics, and computer science were the most requested topics. The majority of respondents concluded that a visionary elective curriculum would make medical students better physicians later on. The study thus provides the first orienting data for the student perspective on visionary content in medical studies, as called for in the “visionary axis” of the graduate profile of the new *NKLM 2.0*.

## Author contributions

- Conceptualization: Eder, Gerhard, Rohr, Salvermoser, Schmidt
- Methodology: Eder, Gerhard, Schmidt
- Validation: Fischer, Dimitriadis
- Formal analysis: Eder, Rohr, Schmidt
- Investigations: Eder, Gerhard, Rohr, Salvermoser, Schmidt
- Resources: Dimitriadis, Fischer
- Data management: Eder, Rohr, Schmidt
- Transcript - preparation manuscript: Eder, Gerhard, Rohr, Salvermoser
- Transcript - review & editing: Dimitriadis, Fischer, Gerhard, Rohr, Salvermoser
- Visualization: Eder, Gerhard, Rohr, Salvermoser
- Supervision: Fischer, Dimitriadis
- Project coordination: Gerhard, Rohr

(according to CRediT taxonomy, arranged alphabetically)

## Acknowledgements

The authors would like to thank the LMU Munich Student Excellence Program (MeCuM StEP) for funding and in particular the other fellows of the class of 2019: Stephan Berthold, Katharina Eisenhut, Daniel Petersheim, Nicola Schieferdecker, Danmei Zhang. The authors would like to thank Marco Brücke (1997-2020) for his visionary mindset in creating the system transcendent axis “Der/Die Visionär*in” and the AG Medizinische Ausbildung der Bundesvertretung der Medizinstudierenden in Deutschland e.V. (bvmd) for the elaboration of the corresponding position paper. S.O. Rohr thanks the German National Academic Foundation for its support. 

## Competing interests

The authors declare that they have no competing interests. 

## Supplementary Material

Primary data
