# Supplementary material for: Thinking outside the box: students positive about visionary elective curricula in medical school
Source: GMS J Med Educ. 2021 Nov 15;38(7):Doc119. doi: 10.3205/zma001515 (PMC8675382; doi:10.3205/zma001515)
Supplement: Primary data [file JME-38-7-119-s-001.pdf]

Attachment 1: Primary data

Table 1: Study progress of the participating medical students at the time of the survey. The modules denote the respective clinical semesters at LMU.

Feature	Frequency	Percentage
**Study progress**		
Preclinical	63	26,7
Module 1	28	11,9
Moduel 2	16	6,8
Module 3	31	13,1
Module 4	36	15,3
Module 5	46	19,5
Module 6	4	1,7
Practical year	1	0,4
Semester off	2	0,8
NS	9	3,8
**Sex**		
Male	76	32,2
Female	158	66,9
NS	2	0,8

(NS = not stated, *N* = 236)

Table 2: Further subject reference of medical students at LMU at the time of the survey with regard to a previous or second degree or previous professional training.

Feature	Frequency	Percentage
**Previous studies**		
Yes	41	17,4
No<br>NS	183<br>12	77,5<br>5,1
**Secondary studies (current)**		
Yes	6	2,5
No<br>NS	207<br>23	87,7<br>9,7
**Subjects**<br>Biochemistry	1	0,4
Bioinformatics	1	0,4
Economy	1	0,4
Physics	1	0,4
Art History	1	0,4
Dentistry	1	0,4
**Secondary studies (Interest)**		
Yes	23	9,7
No<br>NS	195<br>18	82,6<br>7,6
**Professional training**		
Yes	50	21,2
No<br>NS	168<br>18	71,2<br>7,6
**Subjects**		
Nurse	8	3,4
paramedic	28	11,9
Medical-technical assistance	1	0,4
Medical-pharmaceutical assistance	1	0,4
Other	12	5,1

(NS = not stated)

Table 3: Responses of participating medical students at LMU to the question "In your opinion, to what extent is creative/interdisciplinary/critical thinking currently promoted by the study of human medicine?"

**Likert**	**Creative Thinking**	**Interdisciplinary Thinking**	**Critical Thinking**
1	64 (27%)	11 (5%)	34 (14%)
2	81 (34%)	32 (14%)	43 (18%)
3	34 (14%)	54 (23%)	41 (17%)
4	32 (14%)	62 (26%)	56 (24%)
5	16 (7%)	60 (25%)	45 (19%)
6	4 (2%)	13 (6%)	11 (5%)
NS	5 (2%)	4 (2%)	6 (3%)
Total	236	236	236

(Question type: 6-point Likert scale, 1 = not encouraged, 6 = very encouraged, NS = not stated, *N* = 236)

Table 4: Responses from participating medical students at LMU to the question "To what extent do you think creative/interdisciplinary/critical thinking would currently be enhanced by a visionary elective curriculum?"

**Likert**	**Creative Thinking**	**Interdisciplinary Thinking**	**Critical Thinking**
1	5 (2%)	4 (2%)	5 (2%)
2	18 (8%)	9 (4%)	9 (4%)
3	17 (%)	14 (6%)	11 (5%)
4	62 (7%)	40 (17%)	56 (24%)
5	76 (26%)	81 (34%)	88 (37%)
6	42 (18%)	68 (29%)	54 (23%)
NA	16 (7%)	20 (8%)	13 (6%)
Total	236	236	236

(Question type: 6-point Likert scale, 1 = not encouraged, 6 = very encouraged, NS = not stated, *N* = 236)

Table 5: Responses from participating medical students at LMU to the question "Please indicate the extent to which the following topics are currently covered by the human medicine curriculum."

**Likert**	**Philo**	**CS**	**GH**	**Lit**	**Eco**	**Poli**	**Stat**	**Bio**
1	81	63	36	173	101	127	18	6
2	74	86	67	42	87	58	29	8
3	30	39	52	7	18	21	53	20
4	31	21	45	3	10	15	59	41
5	7	10	19	1	4	2	49	87
6	1	2	4	0	1	0	15	57
NS	12	15	13	10	15	13	13	17
Total	236	236	236	236	236	236	236	236

(Question type: 6-point Likert scale, 1 = weakly covered, 6 = strongly covered, NS = not specified, N = 236; Philo = philosophy, CS = computer science, GH = global health, Lit = literary studies, Eco = economics, Poli = political science, Stat = statistics, Bio = biology).

Table 6: Responses from participating medical students at LMU to the question "What topics would you want to address in a visionary elective curriculum?"

**Likert**	**Philo**	**CS**	**GH**	**Lit**	**Eco**	**Poli**	**Stat**	**Bio**
1	58	38	13	76	24	25	48	36
2	26	27	15	41	30	25	40	35
3	22	29	22	20	32	20	47	38
4	39	41	42	33	35	47	46	55
5	31	50	60	25	61	53	19	31
6	37	33	64	22	40	50	16	22
NS	23	18	20	19	14	16	20	19
Total	236	236	236	236	236	236	236	236

(Question type: 6-point Likert scale, 1 = weakly covered, 6 = strongly covered, NS = not specified, N = 236; Philo = philosophy, CS = computer science, GH = global health, Lit = literary studies, Eco = economics, Poli = political science, Stat = statistics, Bio = biology).

Table 5: Responses from participating medical students at LMU to the question "Do you think a project like the visionary elective curriculum makes medical students better doctors?"

Likert	Frequency	Percentage
1	51	22,6
2	75	33,3
3	58	25,8
4	15	6,7
5	17	7,6
6	9	4,0
NS	11	4,7
Total	236	100

(Question type: 6-point Likert scale, 1 = not encouraged, 6 = very encouraged, NS = not stated, *N* = 236)

Table 6: Responses from participating medical students at LMU to the optional question "Do you think a project like the visionary elective curriculum makes medical students better doctors?"

	Wording	Frequency
Yes	Expands non-medical general knowledge/horizon	37
	Expands non-medical specific knowledge	2
	Promotes critical/creative thinking	17
	Matures personality	1
	Not assignable to exact performance	3
No	Only personal initiative allows students to become better physicians	1
Undecided	Only dedicated students benefit from an elective curriculum	2
NS	/	4
Total	/	67

(Question type: free text response, *N* = 67)

Table 7: Parametric comparison between preclinical and clinical students on the question "Would you want to participate in a visionary elective curriculum?"

		N	Mean	Std. deviation	Std. error	95% confidence interval for the mean value		Mini-mum	Maxi-mum	Variance between components
						Upper limit	Lower limit			
Preclinical		56	4,86	1,052	,141	4,58	5,14	2	6	
Clinical		154	4,35	1,532	,123	4,11	4,59	1	6	
Total		210	4,49	1,435	,099	4,29	4,68	1	6	
Model	Fixed effects			1,421	,098	4,29	4,68			
	Random effects				,270	1,06	7,91			,104

(A) Descriptive statistics, question type: 6-point Likert scale, 1 = strongly disagree, 6 = strongly agree, N = 210

	Sum of squares	df	Mean of squares	F	Significance
Between the groups	10,535	1	10,535	5,218	,023
Within groups	419,922	208	2,019		
Total	430,457	209			

(B) Single factorial ANOVA

Table 8: Parametric comparison between students in the preclinical and clinical study sections. 'Should interfaces of medicine with other non-medical specialties be addressed in human medical studies?'

		N	Mean	Std. deviation	Std. error	95% confidence interval for the mean value		Mini-mum	Maxi-mum	Variance between components
						Upper limit	Lower limit			
Preclinical		59	1,10	,305	,040	1,02	1,18	1	2	
Clinical		156	1,22	,414	,033	1,15	1,28	1	2	
Total		215	1,19	,390	,027	1,13	1,24	1	2	
Model	Fixed effects			,387	,026	1,13	1,24			
	Random effects				,061	,41	1,96			,005

(A) Descriptive statistics, question type: 6-point Likert scale, 1 = strongly disagree, 6 = strongly agree, N = 215

	Sum of squares	df	Mean of squares	F	Significance
Between the groups	,579	1	,579	3,854	,051
Within groups	31,980	213	,150		
Total	32,558	214			

(B) Single factorial ANOVA
